# Mitochondrial Fission in Nickel Nanoparticle-Induced Reproductive Toxicity: An In Vitro GC-1 Cell Study

**DOI:** 10.3390/nano14080689

**Published:** 2024-04-17

**Authors:** Hanyue Zheng, Geyu Liang, Chunliu Guan, Lin Liu, Jiahui Dong, Jinshun Zhao, Meng Tang, Lu Kong

**Affiliations:** 1 Key Laboratory of Environmental Medicine and Engineering, Ministry of Education, School of Public Health, Southeast University, Nanjing 210009, China; hanyue_zheng@seu.edu.cn (H.Z.); gyliang@seu.edu.cn (G.L.); 220223680@seu.edu.cn (C.G.); 220193592@aa.seu.edu.cn (L.L.); 220203815@seu.edu.cn (J.D.); tm@seu.edu.cn (M.T.); 2 Allegheny Health Network Cancer Institute, Pittsburgh, PA 15212, USA; zhaojinshun@nbu.edu.cn

**Keywords:** mitochondrial division, nickel nanoparticle, apoptosis, reproductive toxicity, GC-1 cell, environmental pollutant

## Abstract

Reproductive disorders and declining fertility rates are significant public health concerns affecting birth rates and future populations. Male infertility, often due to spermatogenesis defects, may be linked to environmental pollutants like nickel nanoparticles (Ni NPs). Ni NPs are extensively utilized across different industries. Nevertheless, their potential adverse effects cannot be overlooked. Previous studies have linked the reproductive toxicity induced by Ni NPs with disturbances in mitochondrial function. Mitochondrial division/fusion dynamics are crucial to their proper function, yet little is known about how Ni NPs perturb these dynamics and whether such perturbation contributes to the impairment of the male reproductive system. Herein, we demonstrated that the exposure of Ni NPs to the mouse-derived spermatogonia cell line (GC-1 cells) triggered DRP1-mediated mitochondrial division and the enhanced impairment of mitochondria, consequently promoting mitochondria-dependent cell apoptosis. Notably, both the mitochondrial division inhibitor (Mdivi-1) and lentiviral-transfected cells with low expression of Dnm1l-DK in these cells could mitigate the toxic effects induced by Ni NPs, pointing to the potential role of mitochondrial dynamics in Ni NP-induced reproductive toxicity. Collectively, our work contributes to the understanding of the mechanisms by which Ni NPs can impact male reproductive function and identifies mitochondrial division as a potential target for intervention.

## 1. Introduction

Reproductive health problems, including infertility, result in significant societal costs [1]. Among infertile couples, approximately 50% of cases are attributable to the male partner [2,3], causing significant marital and psychosocial stress. Different lifestyles, together with various environmental pollutants and occupational factors, may impair male reproductive function [4,5]. Owing to the electronic properties of nickel and emergent nanomaterials, nickel nanoparticles (Ni NPs) possess unique properties and are widely utilized in various industries, such as biomedicine, electronics, chemicals, aerospace, and so on [6,7]. However, the potential hazards cannot be ignored and it is necessary to responsibly balance the benefits and risks of nanomaterials. Earlier research has focused on the negative impacts of Ni NPs on the respiratory and cardiovascular systems, with limited attention given to other human systems [7,8,9]. Evidence is mounting that Ni NPs can have negative impacts on reproductive health, including disordered reproductive hormone levels, the impairment of sperm production and function, abnormalities in sperm morphology, and damage to the testicular structure [10,11]. Existing research indicates that the reproductive toxicity induced by Ni NPs is linked to mitochondrial harm and mitochondria-dependent apoptosis via oxidative stress [12]. As we know, mitochondrial dynamics are fundamentally important in maintaining the healthy function of mitochondria in cells [13,14,15]. Mitochondria can merge and share their genetic and protein materials through fusion and division processes [16]. Mitochondrial division is characterized by the splitting of a single mitochondrion into two daughter mitochondria, while mitochondrial fusion is a process in which two mitochondria collide end-to-end and eventually merge into a single mitochondrion [17]. During the process of mitochondrial division, the outer membrane protein, mitochondrial division 1 (FIS1), and cytoplasmic dynamin-related protein 1 (DRP1) mediate the division event. During mitochondrial fusion, the mitofusin 1/2 (MFN1/2) proteins on the outer mitochondrial membrane and the optic atrophy 1 (OPA1) protein on the inner mitochondrial membrane facilitate the process [18,19]. 

Our recent study revealed that Ni NPs negatively affected the reproductive system in rats, mice, and Caenorhabditis elegans. Notably, the male reproductive system exhibited greater susceptibility compared to the female reproductive system [10,20]. Meanwhile, we also discovered that the reproductive toxicity induced by Ni NPs in rat primary spermatogenic cells was closely related to cell apoptosis, which could be mediated through the LOC102551356/IGF-BP3/p53 axis and mitochondria-dependent apoptosis pathway [21]. In addition, the ultrastructure of the testis tissue from male animals further manifested the structural damage of mitochondria, such as demonstrating the vacuolation of mitochondria, the blurring or even the disappearance of mitochondrial cristae, increased numbers and decreased volumes of mitochondria, etc. [20]. Therefore, it can be inferred that mitochondria can serve as the target organelles for Ni NP-induced germ cell apoptosis and reproductive system damage via affecting the normal mitochondrial segregation of spermatogenic cells. 

Utilizing the mouse-derived spermatogonia cell line (GC-1 cells) as our experimental model, we aimed to unravel the potential reproductive toxicity in males caused by Ni NPs and to uncover the underlying molecular mechanisms that govern mitochondrial division in the context of this toxicity. We employed a dual-pronged approach involving the application of mitochondrial division inhibitor 1 (Mdivi-1) and the lentiviral-mediated overexpression of Dnm1l (the gene name of the DRP1 protein) to elucidate the regulatory mechanisms of mitochondrial division. Mdivi-1 acts as a specific inhibitor of DRP1, a indispensable protein for the fission of mitochondria, while Dnm1l, a dominant-negative mutant form of DRP1, effectively obstructs mitochondrial division. By manipulating these factors, we sought to reverse the detrimental effects of Ni NPs on mitochondrial dynamics and gauge their subsequent impacts on reproductive toxicity. 

The findings of this study, which yielded significant insights, are poised to become a valuable reference for future research aimed at understanding the harmful effects of Ni NPs regarding reproductive health. Our work transcends the boundaries of the potential exposure routes, providing a comprehensive perspective on the issue. Concurrently, this research provides potential targets of Ni NPs and offers a scientific basis for the development of strategies to mitigate the reproductive risks associated with Ni NP exposure, thereby safeguarding both the environment and the occupational health of populations at risk.

## 2. Materials and Methods

### 2.1. Cell Line

The GC-1 cell line was obtained from the Shanghai Institutes for Biological Sciences, Chinese Academy of Sciences (Shanghai, China). GC-1 cells were cultured in DMEM supplemented with 10% FBS and 1% penicillin–streptomycin and incubated under the conditions of 37 °C and 5% CO_2_. Cells within the logarithmic growth phase, characterized by a cell density of 70% to 80% in the culture dish and demonstrating robust health, were selected for subsequent experimental analysis.

### 2.2. Ni NPs and Their Characterization

Ni NPs (product number: FNiN-80) were obtained from Nano Science and Kunshan City Miyou Technology Co., Ltd., Kunshan, China. The Ni NPs were black powders with purity of 99%, a surface area of no less than 8 m^2^/g, a bulk density ranging from 0.06 to 0.8 g/cm^3^, and an average size of 90 nm. The physical characteristics of the Ni NPs were assessed using scanning electron microscopy (SEM, JEOL, Tokyo, Japan) and transmission electron microscopy (TEM, JEOL, Tokyo, Japan), as detailed in previous studies [10].

### 2.3. Preparation of Ni NP Suspension

Under sterile conditions, a measured amount of Ni NP powder was combined with an appropriate volume of sterile complete medium, followed by ultrasonication for 30 s on ice to prepare the Ni NP suspension. Subsequently, the suspension underwent further sonication for 3 min at 400 W. The resulting suspension was then diluted to the desired concentration for subsequent experiments.

### 2.4. In Vitro Cytotoxicity Assay

GC-1 cells were employed to assess the cytotoxicity of Ni NPs via the CCK-8 assay (Nanjing Vazyme Biotech Co., Ltd., A311-01, Nanjing, China). Briefly, the GC-1 cells were seeded in 96-well plates (*n* = 3) at a density of 8 × 10^3^ cells/mL per well and cultured with Dulbecco’s modified Eagle’s medium (DMEM, Gibco, Waltham, MA, USA) supplemented with 10% FBS and 1% penicillin at 37 °C in a 5% CO_2_ humidified environment. Ni NPs were diluted in DMEM at the final concentrations of 25, 50, and 100 μg/mL and then added to each well to incubate with the cells. After a 24 h incubation period, a CCK-8 working solution was added, following by incubation for another 1.5 h. The relative optical density (OD) of each well was then measured at a wavelength of 450 nm using an automatic microplate reader. The mean OD from three measurements was obtained in the indicated groups, with the median inhibitory concentration (IC50) fitting curve obtained using the GraphPad Prism 8.0.2 software.

### 2.5. Analysis of Cell Apoptosis

The cell apoptosis analysis was performed using the Annexin V-FITC Apoptosis Detection Kit (Nanjing Vazyme Biotech Co., Ltd., A211-01/02, Nanjing, China), following the manufacturer’s protocol. Briefly, GC-1 cells were seeded in six-well plates at a density of 1.8 × 10^5^ cells/mL per well and incubated separately with the Ni NP suspension at concentrations of 0, 25, 50, and 100 μg/mL. Subsequently, the cells were trypsinized with 0.25% trypsin (without EDTA), washed three times with pre-chilled phosphate-buffered saline, and centrifugated at 1500 rpm for 5 min. The cells were resuspended in 500 μL binding buffer and stained with 5 μL FITC and 5 μL PI in the dark for 15 min. Finally, cell apoptosis was detected by flow cytometry (FACSCalibur, BD Biosciences, San Jose, CA, USA) and then analyzed using the FlowJo_V10 software.

### 2.6. Ultrastructures of Cells

GC-1 cells were treated with or without 100 μg/mL Ni NP suspension for 24 h. Subsequently, the cells were trypsinized with 0.25% trypsin (without EDTA) and centrifugated at 1500 rpm for 5 min. After being washed three times with PBS solution, the cells were fixed in 2.5% glutaraldehyde and then centrifugated, washed again, and finally fixed in 1% osmic acid fixative for 2 h. Sections with an approximately 70 nm thickness were cut and stained using the uranyl acetate–lead citrate double staining method on a copper grid, and the ultrastructures of the cells were observed under TEM (H-7650C, Hitachi, Japan).

### 2.7. Mitochondrial Morphology

C-1 cells were seeded in confocal dishes at a density of 6 × 10^4^ cells/mL for 24 h. Subsequently, they were treated with the Ni NP suspension at the concentrations of 0, 25, 50, and 100 μg/mL for another 24 h, respectively. The Mito Tracker@ Red CMXRos probe powder (Yi Sheng Biotechnology (Shanghai) Co., Ltd., M7512, Shanghai, China) was dissolved to 1 mM in dimethyl sulfoxide and then diluted to 100 nM working solution in a complete culture medium. After the addition of 1 mL working solution to each dish, the cells were incubated at 37 °C for 40 min in the dark. Afterward, they were washed twice with PBS and resuspended in 1 mL complete culture medium. The morphology of the mitochondria (Ex = 579 nm, Em = 599 nm) was observed under confocal laser scanning microscopy (FV3000, Olympus, Tokyo, Japan).

### 2.8. Determination of Reactive Oxygen Species (ROS)

Intracellular ROS generation was determined by the dichloro-dihydro-fluorescein diacetate (DCFH-DA) assay (Beyotime Biotechnology, S0033S, Shanghai, China). GC-1 cells were seeded in six-well plates at a density of 1.8 × 10^5^ cells/mL per well and treated separately with the Ni NP suspension for 24 h. The DCFH-DA probe was diluted to 10 µM with serum-free DMEM medium at a ratio of 1:1000, and 1 mL diluted solution was then added to each well and incubated for 30 min at 37 °C in the absence of light. The cells were washed thrice with PBS and then examined under an inverted fluorescence microscope (Axiovert A1, ZEISS, Oberkochen, Germany). The fluorescence intensities (Ex = 488 nm, Em = 525 nm) were analyzed using the ImageJ (1.52a) software.

### 2.9. Determination of Mitochondrial Reactive Oxygen Species (MtROS)

GC-1 cells were seeded in 12-well plates at a density of 6 × 10^4^ cells/mL per well and exposed to the Ni NP suspension for 24 h. Then, 5 mM MitoSOX Red Mitochondrial Superoxide Indicator stock solution (Yi Sheng Biotechnology (Shanghai) Co., Ltd., 40741ES50, Shanghai, China) was diluted to 5 µM with PBS at a ratio of 1:1000. After 10 min incubation with 1 mL working solution per well in a shaking incubator, the fluorescence intensity (Ex = 510 nm, Em = 580 nm) of the cells was observed, and photographs were captured under an inverted fluorescence microscope (Axiovert A1, ZEISS, Germany).

### 2.10. Measurement of Mitochondrial Membrane Potential (MMP)

An MMP detection kit (JC-1) was utilized to monitor the alterations in MMP (Beyotime Biotechnology, C2003S, Shanghai, China), following the manufacturer’s instruction. Briefly, GC-1 cells were seeded in six-well plates at a density of 1.8 × 10^5^ cells/mL per well and subsequently exposed to the Ni NP suspension for 24 h. Following 1 h of incubation with 1 mL JC-1 staining solution per well in a shaking incubator maintained at 37 °C, the cells underwent a washing process with 4 °C precooled JC-1 staining buffer, which was repeated twice. Subsequently, the cells were resuspended in 1 mL complete culture medium in each well. Finally, the red fluorescence of J-aggregates, reflecting higher mitochondrial potential (Ex = 585 nm, Em = 590 nm), and the green fluorescence of J-monomers, indicating lost membrane potential (Ex = 514 nm, Em = 529 nm), were examined using inverted fluorescence microscopy (Axiovert A1, ZEISS, Germany). The fluorescence intensity was quantified with the ImageJ (1.52a) software.

### 2.11. Measurement of Adenosine Triphosphate (ATP)

The intracellular levels of ATP were quantified using an ATP Determination Kit (Beyotime Biotechnology, S0026B, Shanghai, China). In short, 1.8 × 10^5^ cells per well were seeded in six-well plates for 24 h and then incubated for 24 h. Following this, the cells were treated with the Ni NP suspension. After 24 h exposure, cells were treated with 0.25% trypsin (without EDTA) to detach them, and then harvested by centrifugation at 12,000 rpm for 5 min. The cells were rinsed thrice with PBS and disrupted in 200 μL ATP assay buffer. The ATP standard solution was diluted with ATP detection lysate into standard substances with different concentration gradients for the determination of the standard curve. Subsequently, 20 μL supernatant from the cell lysate was combined with 100 μL reaction solution, and the fluorescence was recorded. The level of ATP in the samples was calculated through the standard curve and expressed as a relative light unit (RLU) value using a multifunctional microplate reader (Tristar 5 LB942, Berthold, Bad Wildbad, Germany).

### 2.12. Western Blot Analysis

Western blot analysis was conducted to evaluate the expression of proteins associated with mitochondrial function and apoptotic pathways. After a 24 h incubation period, whole protein extracts were obtained from the GC-1 cells. The protein content was then measured and quantified using the bicinchoninic acid (BCA) protein assay (Beyotime Biotechnology, P0009, Shanghai, China). Protein samples were resolved by sodium dodecyl sulfate–polyacrylamide gel electrophoresis (SDS-PAGE) and subsequently transferred to a nitrocellulose membrane (PALL, Port Washington, NY, USA). The membrane was blocked with 5% non-fat milk for 2 h at ambient temperature before being probed with primary antibodies diluted at 1:1000 (Boster, Pleasanton, CA, USA) at 4 °C overnight. After three rounds of washing with Tris-buffered saline containing Tween-20 (TBST), the membrane was further incubated with secondary antibodies diluted at 1:5000 (Boster, Pleasanton, CA, USA) for 2 h at ambient temperature. After being washed four times with TBST, the membrane was developed with a chemiluminescence solution (Thermo, Waltham, MA, USA) to illuminate the bands, which were then captured using a chemiluminescence automatic image analysis system (Tanon, Shanghai, China). The gray value of the protein blot was analyzed via the ImageJ SAAinc software (1.52a).

### 2.13. Determination of Optimal Concentration of Inhibitor Mdivi-1

To ascertain the most effective concentrations of Mdivi-1, cell viability was assessed using the CCK-8 assay. Initially, GC-1 cells were seeded in 96-well plates at a density of 8 × 10^3^ cells/100 μL per well and allowed to grow for 24 h. The existing growth medium was then substituted with fresh medium containing varying concentrations of Mdivi-1, specifically 0, 5, 10, 15, 20, and 25 μM, and the cells were further incubated for another 3 h. Following the addition of 100 μg/mL Ni NPs for 24 h, the CCK-8 solution was added and the mixture was incubated in the dark for 1.5 h. Thereafter, the corresponding absorbance value was recorded using a microplate reader.

### 2.14. Experimental Group

C-1 cells were divided and then incubated with the culture medium (control group), 15 μM Mdivi-1 (Mdivi-1 group), 100 μg/mL Ni NPs (Ni NP group), or 15 μM Mdivi-1 + 100 μg/mL Ni NPs (Ni NP+Mdivi-1 group).

### 2.15. Lentiviral Transduction of GC-1 Cells and Screening of Stable Strains

The lentiviral packaging system was obtained from Shanghai Jikai Gene Medical Technology Co., Ltd., Shanghai, China. and utilized a vector named GV493. This vector is designed with the structural elements hU6-MCS-CBh-gcGFP-IRES-puromycin, allowing for the expression of the enhanced green fluorescent protein (EGFP) under the control of the HitransG A infection enhancer. The cloning process involved the insertion of the Dnm1l shRNA sequences into the MCS of the GV493 vector, following a series of molecular biology techniques including restriction enzyme digestion, ligation, and transformation into competent cells. The recombinant plasmids were then verified through PCR amplification and Sanger sequencing to ensure the correct insertion of the target sequences.

GC-1 cells were seeded in 96-well plates for 24 h and then cultured with different MOIs (0, 10, 50, and 100) of lentivirus for 72 h; the green fluorescence intensity of EGFP (Ex = 488 nm, Em = 507 nm) was observed under inverted fluorescence microscopy to judge the cell transduction efficiency. If it was more than 80%, together with a good cell growth condition, it was selected as the optimal MOI for transduction. If the transduction efficiency and cell growth status were similar, a lower MOI was selected as the optimal MOI for lentiviral transduction. Thereafter, the cells were separately transduced with slow lentiviral packaging with low Dnm1l expression (Dnm1l-KD group) and virus packaging with low Dnm1l expression as a negative control group (Dnm1l-NC group). Meanwhile, the cells incubated with 100 μg/mL Ni NPs comprised the Ni NP group, and those without transduction and Ni NPs were used as the control group.

### 2.16. Statistical Analysis

Data were derived from three separate experiments. Results are depicted as the mean ± standard deviation. A T test was employed to evaluate the statistical significance between two distinct groups, and a one-way analysis of variance (ANOVA) was conducted for the comparison of multiple groups using the GraphPad Prism 8.0.2 software. A *p* value of less than 0.05 was considered to indicate a statistically significant result.

## 3. Results

### 3.1. Characterization of Ni NPs

The Ni NPs observed through SEM and TEM images appeared spherical in shape, with a size range of 30 to 100 nm in diameter and an average size of 90 nm. Some slight agglomeration was also observed. In the dispersion, the particle size of the Ni NPs at a concentration of 5 Lg mL^−1^ ranged from 260 to 725 nm, with a peak size of approximately 444 nm. At a concentration of 12.5 lg mL 21, the average particle size distribution ranged from 400 to 879 nm, with a peak size of about 522 nm. On the other hand, the Ni NPs had an average size distribution of 3.34 ± 0.67 µm and did not exhibit any agglomeration. According to the findings, the nickel nanoparticles with a diameter of 90 nm appeared as uniformly shaped spheres, but also showed some instances of agglomeration. The degree of particle agglomeration was observed to be affected by the concentration of particles in the dispersion. The results have been extensively documented in our earlier experimental research [10].

### 3.2. Study on Toxicity of Ni NPs on GC-1 Cells

**Figure 1:**
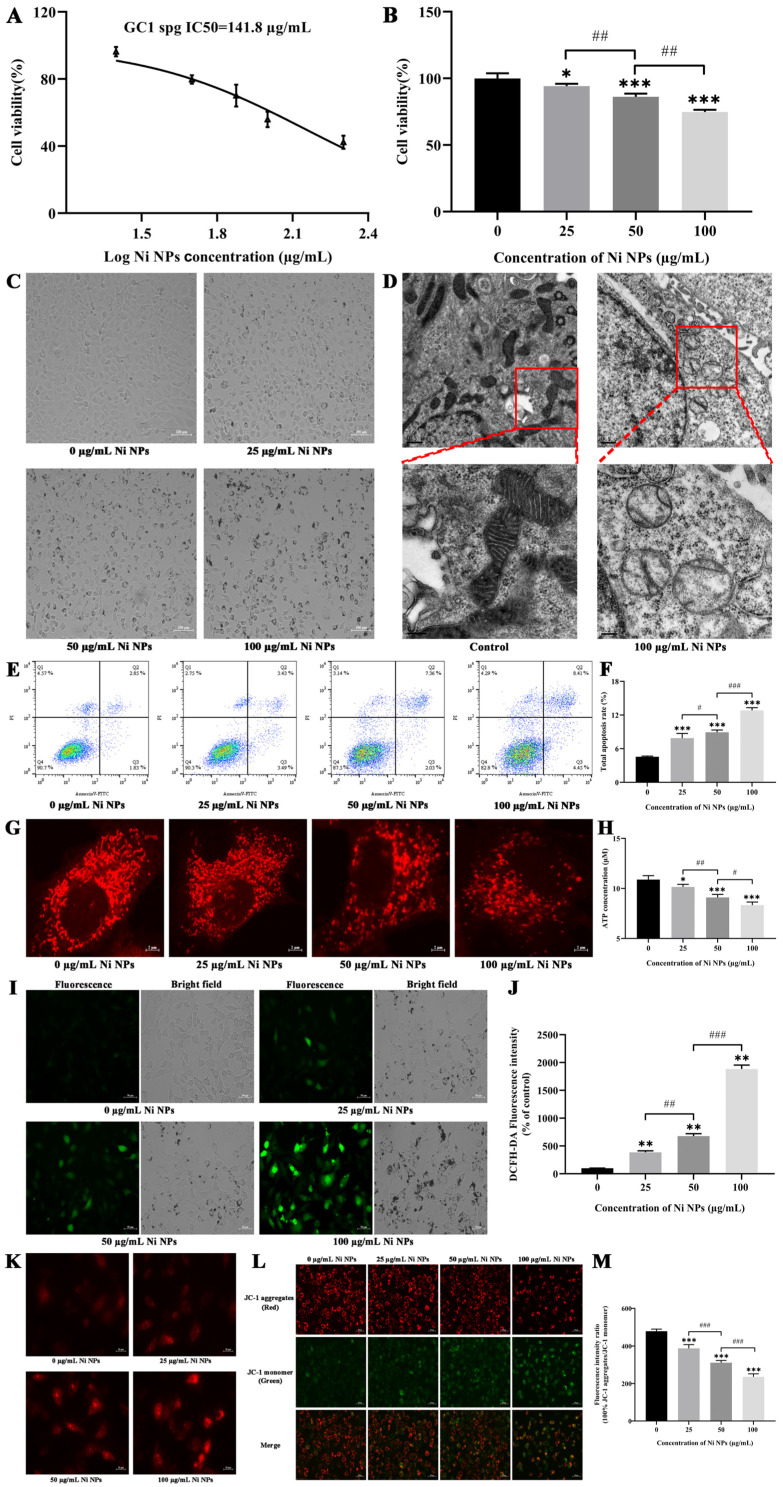
Effects of Ni NPs on the cell viability, cell morphology, ultrastructure, apoptosis rate, damage of mitochondrial structures, ATP energy production, intracellular ROS, MtROS, and MMP of GC-1 cells. (**A**) Curve fitting of IC50 of GC-1 cells at 24 h. (**B**) Viability of GC-1 cells after 24 h incubation with different concentrations of Ni NPs. (**C**) Morphology of GC-1 cells (100×) under light microscopy after 24 h incubation with different concentrations of Ni NPs. (**D**) Image of ultra-structure of GC-1 cells under transmission electron microscopy after 24 h incubation with or without 100 μg/mL Ni NPs (scale bar: 0.5 µm for the upper images and 0.2 µm for the locally enlarged images of the upper ones). (**E**,**F**) Dot plot representing four quadrant images and histogram of apoptosis rate in GC-1 cells exposed to different concentrations of Ni NPs for 24 h. Q1, necrotic cells; Q2, late apoptosis; Q3, early apoptosis; Q4, normal cells. (**G**) Mitochondrial morphology of GC-1 cells (600×) exposed to different concentrations of Ni NPs for 24 h. (**H**) Histogram of ATP content in GC-1 cells exposed to different concentrations of Ni NPs for 24 h. (**I**,**J**) Changes in ROS in GC-1 cells exposed to different concentrations of Ni NPs. (**K**) Changes in MtROS in GC-1 cells exposed to different concentrations of Ni NPs for 24 h. (**L**,**M**) Changes in MMP in GC-1 cells exposed to different concentrations of Ni NPs for 24 h. (**I**,**L**) Scale bar = 50 μm; (**K**) Scale bar = 20 μm. * *p* < 0.05, ** *p* < 0.01, *** *p* < 0.001, compared with control group. # *p* < 0.05, ## *p* < 0.01, ### *p* < 0.001, compared between two groups.

Using GC-1 cells as the research objects, the cell viability, cell morphology, intracellular apoptosis, mitochondria ultrastructure, ATP, ROS, MtROS, and MMP were observed after 24 h incubation with different concentrations of Ni NPs. The IC_50_ of the Ni NPs was 141.8 µg/mL for GC-1 cells (Figure 1A), and the viability of GC-1 cells was reduced in a dose-dependent manner upon exposure to Ni NPs (*p* < 0.05) (Figure 1B). Consistent with the decreased cell viability, the cell morphology under light microscopy directly confirmed the toxicity of the Ni NPs towards the GC-1 cells. There was sufficient evidence that the morphology of the GC-1 cells gradually became rounded and broken, the cell growth density decreased, and the intercellular junctions were weakened with the increasing concentration of Ni NPs (Figure 1C). 

It is well known that mitochondria are organelles enclosed by two membranes with cristae. In the present study, the observation of the intracellular ultrastructure under TEM revealed that the morphology of the mitochondria in the GC-1 cells was fragmented when compared to the control group. In particular, a reduced and broken mitochondrial cristae structure was easily visible in the locally enlarged image of the 100 μg/mL Ni NP group (Figure 1D). 

The use of Annexin V-FITC/PI allowed for the identification and quantification of apoptotic and necrotic cells after 24 h incubation of GC-1 cells treated with varying concentrations of Ni NPs. The results presented in Figure 1E,F indicate that exposure to Ni NPs at concentrations of 25 and 100 µg/mL caused a significant increase in early apoptosis when compared to the control group (*p* < 0.05). Furthermore, the percentages of late apoptosis and total apoptosis were also significantly higher, in a dose-dependent manner, when compared to the control group (*p* < 0.05). These results indicate that Ni NPs can induce cell apoptosis and impair reproductive health. 

With the development of high-resolution fluorescent microscopy, it can be observed that mitochondria, known as highly dynamic structures, exhibit significant structural intricacy and diversity, particularly in their inner folded membranes. As shown in Figure 1G, the morphofunctional changes in the mitochondria were observed after treatment for 24 h. For example, the number of mitochondria gradually decreased in a dose-dependent manner, and their morphology was broken into scattered dots; thus, the integrity of the network structure was destroyed. It is suggested that Ni NPs can enhance mitochondrial division in GC-1 cells (Figure 1G). Moreover, the ATP content in GC-1 cells decreased in a dose-dependent manner compared to the control group (*p* < 0.05) (Figure 1H), indicating that the Ni NPs inhibited the intracellular ATP production.

The information described in Figure 1I,J illustrates that the fluorescence intensity of the GC-1 cells increased significantly and the green fluorescence of the GC-1 cells became brighter as the concentration of Ni NPs increased in comparison to the control group (*p* < 0.01, *p* < 0.001), indicating that Ni NPs can cause an increase in the production of ROS in GC-1 cells, and the extent of this effect is dependent on the dosage of Ni NPs. A similar result for MtROS in the GC-1 cells was also detected in the Ni NP group (Figure 1K). 

In this study, it was uncertain whether mitochondria played a role in the induction of apoptosis by Ni NPs. To investigate this further, we utilized JC-1, a fluorescent mitochondrial probe, to evaluate the levels of MMP. We observed that, compared to the control group, the levels of MMP (Figure 1L,M) underwent a dose-dependent reduction in the Ni NP group (*p* < 0.001), indicating that Ni NPs can cause intracellular mitochondrial dysfunction in the genital system.

The data indicated that the Ni NPs caused selective damage to the cellular organelles of the GC-1 cells, resulting in male reproductive toxicity.

### 3.3. Determination of Optimal Conditions for Mdivi-1 and Dnm1l

**Figure 2:**
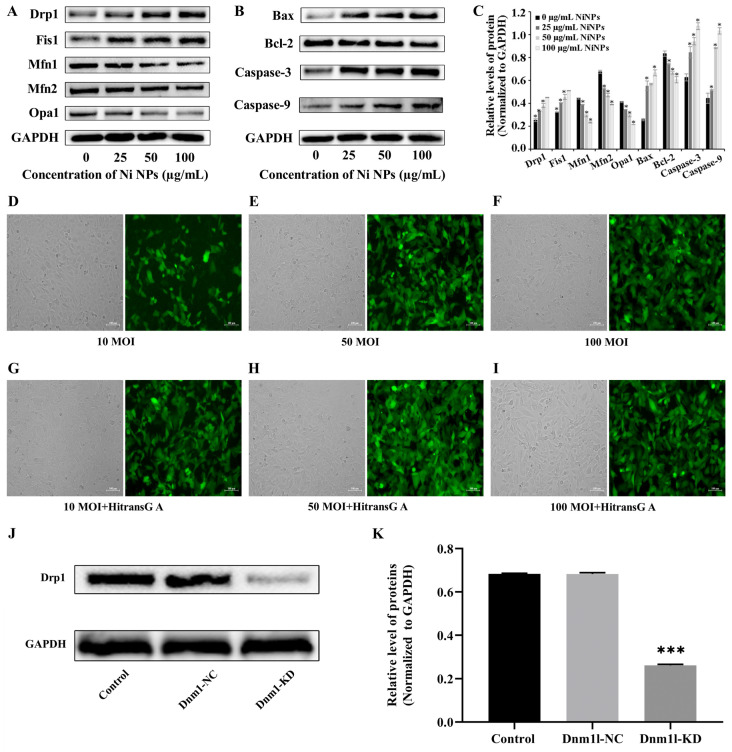
Ni NPs’ inducement of mitochondrial dynamics and apoptosis of GC-1 cells, as well as optimization and validation of best experimental conditions. Representative Western blot and relative expression levels of mitochondria and apoptosis-associated proteins in GC-1 cells exposed to different concentrations of Ni NPs (**A**–**C**) for 24 h. Data in the bar graph represent mean ± SD of three independent measures. (**D**–**I**), Images of lentivirus-transfected GC-1 cells after use of different MOIs of lentivirus transfection with or without additional Hitrans G A infection enhancer for 24 h. (**J**) Representative band of DRP1 protein after 24 h incubation. (**K**), Relative expression of DRP1 protein after 24 h incubation. * *p* < 0.05, *** *p* < 0.001, compared with control group.

To delve deeper into the mechanisms underlying Ni NP-induced apoptosis and to identify potential countermeasures against the deleterious effects of Ni NPs on GC-1 cells, we conducted a Western blot analysis to assess the expression levels of proteins associated with apoptosis and mitochondrial dynamics. Our findings, as illustrated in Figure 2A–C, revealed the significant upregulation of the levels of DRP1, FIS1, BAX, Caspase-9, and Caspase-3 in GC-1 cells exposed to Ni NPs, in comparison to the control group (*p* < 0.05). Concurrently, the notable downregulation of the levels of the Mfn1, Mfn2, Opa1, and Bcl-2 proteins was observed, leading to an increased BAX/Bcl-2 ratio with escalating doses of Ni NPs (*p* < 0.05). These results suggest that Ni NPs may facilitate germ cell apoptosis through the promotion of mitochondrial division, highlighting the need for interventions that can modulate these processes.

With the aim of mitigating the cytotoxic effects of Ni NPs, we introduced Mdivi-1 and a low Dnm1l expression model. By targeting the key regulators of mitochondrial dynamics, we could attenuate the Ni NP-induced apoptotic pathways and preserve the cell viability, thereby offering a potential strategy to counteract the reproductive toxicity associated with Ni NP exposure. We initiated an investigation to identify the optimal concentration of Mdivi-1, a compound known to inhibit dynamin-related protein 1 (DRP1), a key regulator of mitochondrial fission. GC-1 cells were treated with 100 μg/mL Ni NPs in the presence of varying concentrations of Mdivi-1 (0, 5, 10, 15, and 25 μM) for 24 h. The subsequent assessment of the cell viability using the CCK-8 assay indicated a gradual increase in cell viability from 5 μM to 15 μM Mdivi-1, followed by a decline at higher concentrations (*p* < 0.05). This suggests that high concentrations of Mdivi-1 may elicit adverse effects, thereby underscoring the importance of the optimal concentration of 15 μM Mdivi-1 for subsequent experimental endeavors.

Subsequently, we explored the optimal conditions for the establishment of a GC-1 cell model with the reduced expression of Dnm1l, a gene encoding a protein involved in mitochondrial dynamics, by employing lentiviral transfection. The transfection efficiency and fluorescence intensity of the cells were assessed at different multiplicities of infection (MOI), both with and without the addition of the Hitrans G A infection enhancer (Figure 2D–I). The most pronounced fluorescence and the greatesr proportion of fluorescent cells were observed at an MOI of 50 with the enhancer (Figure 2K), thus indicating this condition to be optimal for our studies.

To validate the effectiveness of the lentiviral transfection in reducing Dnm1l expression, a Western blot analysis was conducted. The results, depicted in Figure 2J,H, confirmed the significant reduction in the expression of the DRP1 protein in the Dnm1l-KD group compared to the control group (*p* < 0.05), while no significant change was observed in the Dnm1l-NC group (*p* > 0.05). This validation confirms the utility of the Dnm1l-KD model in further investigations aimed at elucidating the protective mechanisms against Ni NP-induced apoptosis.

In summary, the introduction of Mdivi-1 and the low Dnm1l expression model was a strategic decision based on the observed enhancement in mitochondrial division and apoptosis in GC-1 cells treated with Ni NPs. By targeting the key regulators of mitochondrial dynamics, we aimed to attenuate the Ni NP-induced apoptotic pathways and preserve the cell viability, thereby offering a potential strategy to counteract the reproductive toxicity associated with Ni NP exposure.

### 3.4. Mdivi-1 and Dnm1l Attenuate Ni NP-Induced Cell Apoptosis and Morphofunctional Changes in Mitochondria in GC-1 Cells

**Figure 3:**
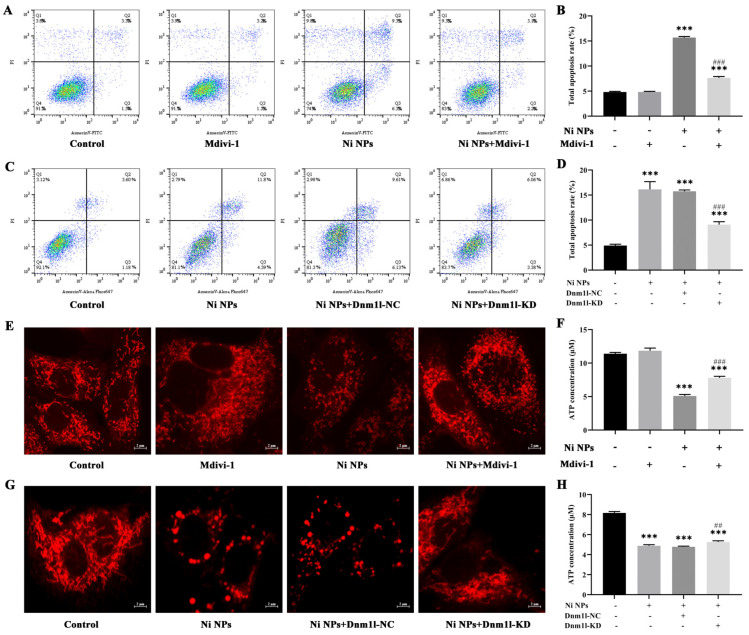
Mdivi-1 and Dnm1l attenuate Ni NP-induced apoptosis rate and damage to mitochondrial structure and ATP energy production in GC-1 cells. (**A**–**D**) Dot plot representing four quadrant images and histogram of apoptosis rate in GC-1 cells exposed to different concentrations of Ni NPs with additional Mdivi-1 (**A**,**B**) or additional Dnm1l (**C**,**D**) for 24 h. Q1, necrotic cells; Q2, late apoptosis; Q3, early apoptosis; Q4, normal cells. (**E**,**G**), Mitochondrial morphology of GC-1 cells (600×) exposure to different concentrations of Ni NPs with additional Mdivi-1 (**E**) or additional Dnm1l (**G**) for 24 h. (**F**,**H**), Histogram of ATP content in GC-1 cells exposed to different concentrations of Ni NPs with additional Mdivi-1 (**F**) or additional Dnm1l (**H**) for 24 h. *** *p* < 0.001, compared with control group. ## *p* < 0.01, ### *p* < 0.001, compared between two groups.

The impact of Mdivi-1 in preventing Ni NP-induced apoptosis was further explored. The rates of early and late apoptosis, as well as total apoptosis, were significantly lower in the group that received Ni NPs along with Mdivi-1, compared to the group that received only 100 μg/mL Ni NPs (*p* < 0.05). This finding is supported by Figure 3A,B, suggesting that Mdivi-1 effectively inhibits Ni NP-induced germ cell apoptosis.

To provide more evidence supporting the involvement of mitochondrial division in Ni NP-triggered apoptosis, the optimal conditions were explored for lentivirus-transfected cells with the low expression of Dnm1l, and further for the construction of a cell model with low expression of Dnm1l (Dnm1l-KD) and its corresponding negative control (Dnm1l-NC). The percentages of late apoptosis and total apoptosis were markedly reduced in the group with Ni NPs and additional Dnm1l-KD, as compared to the group with Ni NPs (*p* < 0.05) (Figure 3C,D). Accordingly, these data imply that Dnm1l-KD attenuates Ni NP-induced germ cell apoptosis. 

The additional Mdivi-1 (Figure 3E) or Dnm1l-KD (Figure 3G) attenuated the Ni NP-induced mitochondrial damage. The most important and meaningful finding was that Mdivi-1 reversed the decrease in Ni NP-induced ATP content in GC-1 cells (Figure 3F), and Dnm1l-KD also significantly suppressed the downregulation of Ni NP-induced ATP content in GC-1 cells (Figure 3H).

**Figure 4:**
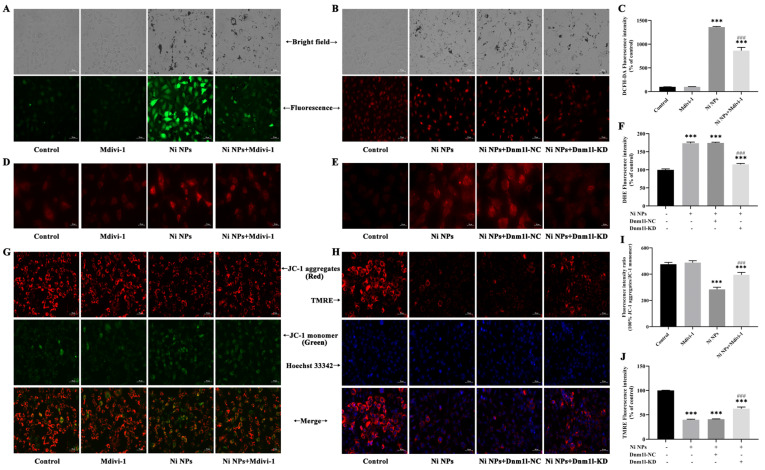
Attenuation of Mdivi-1 and Dnm1l in Ni NP-induced intracellular ROS, mtROS, and MMP in GC-1 cells. (**A**–**C**,**F**) Changes in ROS in GC-1 cells exposed to Ni NPs with additional Mdivi-1 (**A**,**C**) or additional Dnm1l (B,F) for 24 h. (**D**,**E**) Changes in MtROS in GC-1 cells exposed to Ni NPs with additional Mdivi-1 (**D**) or additional Dnm1l (**E**) for 24 h. (**G**–**J**), Changes in MMP in GC-1 cells exposed to Ni NPs with additional Mdivi-1 (**K**,**I**) or additional Dnm1l (**H**,**J**) for 24 h. Scale bar = 50 μm. *** *p* < 0.001, compared with control group. ### *p* < 0.001, compared between two groups.

Meanwhile, our in vitro study of GC-1 cells confirmed that the additional Mdivi-1 significantly decreased the accumulated ROS (Figure 4A,C) and MtROS (Figure 4D) (*p* < 0.001) and raised the levels of intracellular MMP (*p* < 0.001) (Figure 4G,I) as compared with the Ni NP group.

After DHE was oxidized, it entered the cells and was then combined with DNA; thus, red fluorescence emitted from the nucleus could be detected and demonstrated by images of the intracellular fluorescence (Figure 4B) and the fluorescence intensity (Figure 4F). The Ni NP group exhibited a significant increase in the red fluorescence intensity of GC-1 cells (*p* < 0.001), and the red fluorescence intensity could be attenuated by additional Dnm1l-KD (*p* < 0.001) when compared to the control group. The findings suggest that Dnm1l-KD inhibits the accumulation of intracellular ROS induced by Ni NPs in GC-1 cells. Similarly, the combination of Ni NPs with Dnm1l-KD significantly reduced the intracellular MtROS content (*p* < 0.001) (Figure 4E) and increased the intracellular (Figure 4H,J) MMP levels (*p* < 0.001) compared with single Ni NPs.

### 3.5. Mdivi-1 and Dnm1l Attenuate Ni NP-Induced Mitochondria- and Apoptosis-Associated Proteins in GC-1 Cells

**Figure 5:**
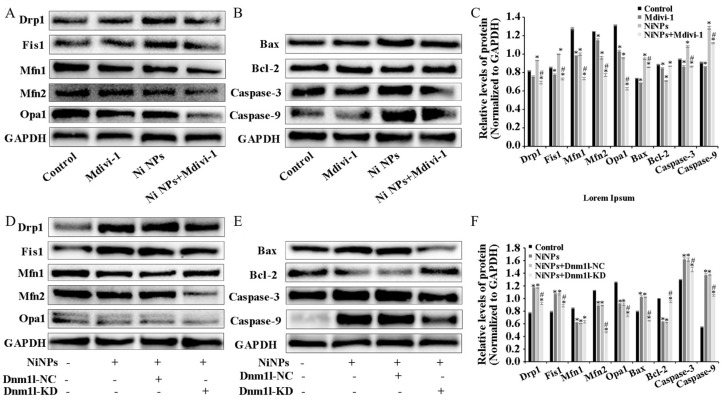
Mdivi-1 and Dnm1l attenuate NP-induced changes in mitochondrial dynamics of GC-1 cells. Notes: A, representative Western blot and relative expression levels of mitochondria- and apoptosis-associated proteins in GC-1 cells exposed to different concentrations of Ni NPs with additional Mdivi-1 (**A**–**C**) or additional Dnm1l (**D**–**F**) for 24 h. Data in bar graph represent mean ± SD of three independent measures. * *p* < 0.05, ** *p* < 0.01 compared with control group. # *p* < 0.05 compared between two groups.

These results suggest that Mdivi-1 can successfully inhibit the enhancement in mitochondrial division in germ cells induced by Ni NPs and decrease the occurrence of apoptosis. Mdivi-1, along with the Ni NPs, led to a notable decrease in the upregulation of the intracellular expression of the DRP1, FIS1, BAX, Caspase-9, and Caspase-3 proteins, compared to single Ni NPs. Moreover, it helped in the restoration of the Bcl-2 protein expression that was downregulated by the Ni NPs, improving the significantly decreased ratio of BAX/Bcl-2 in the GC-1 cells (*p* < 0.05) (Figure 5A–C). 

Consistent with the Mdivi-1 results, Dnm1l also confirmed the involvement of mitochondrial division in the apoptosis induced by Ni NPs in GC-1 cells. Compared with the single Ni NPs, the additional Dnm1l-KD with Ni NPs significantly downregulated the intracellular expression levels of the DRP1, FIS1, BAX, Caspase-9, and Caspase-3 proteins and upregulated the expression level of the Bcl-2 protein, thereby decreasing the ratio of BAX/Bcl-2 (*p* < 0.05) (Figure 5D–F). The results indicate that the low expression of Dnm1l inhibits germ cell apoptosis and alleviates the cytotoxic effects through inhibiting Ni NP-induced mitochondrial division. Collectively, these data demonstrate that Ni NP exposure causes higher levels of ROS and mtROS, leading to mitochondrial damage; a decrease in ATP and MMP; the downregulation of the DRP1, FIS1, BAX, Caspase-9, and Caspase-3 proteins; and the upregulation of the Mfn1, Mfn2, Opa1, and Bcl-2 proteins, consequently inducing germ cell apoptosis. In particular, the Ni NPs induced mitochondrial-mediated cell apoptosis that could be ameliorated by the addition of the inhibitor Mdivi-1 and lentiviral-transfected cells with the low expression of Dmn1l.

## 4. Discussion

The study found that mitochondria may be the key intracellular targeting organelles in the process of germ cell apoptosis induced by Ni NPs. Mitochondrial division may play a role in regulating the toxic effects on the male reproductive system caused by Ni NPs [22]. The homeostasis between mitochondrial division and fusion (mitochondrial dynamics) determines the formation of mitochondrial networks, and it is critical in regulating the mitochondrial size, shape, and number and maintaining homeostasis [23]. Mitochondrial dysfunction is mainly manifested by the overproduction of ROS, the depletion of ATP, the destruction of MMPs, and increased apoptosis [24]. ATP is the only universal energy currency in cells [25]. MMPs are produced from the mitochondrial respiratory chain, which catalyzes a sequence of chemical reactions including redox reactions, reflecting the performance of the mitochondrial electron transport chain [26]. Studies have shown that decreased MMP levels lead to the release of pro-apoptotic factors from mitochondria, thereby activating the Caspase cascade, leading to the occurrence and development of apoptosis. The mitochondrial apoptotic pathway is initiated by Caspase-9, which cleaves and activates Caspase-3. Caspase-3 is a crucial executor of apoptosis and is responsible for cleaving various proteins involved in the process. Bcl-2 and Bax are members of the Bcl-2 family, responsible for regulating mitochondrial or intrinsic apoptotic pathways [27,28,29].

Numerous investigations have revealed that Ni NPs can induce male reproductive toxicity [30]. These nanomaterials, owing to their enhanced penetrative capabilities, are adept at traversing the blood–testis barrier, inflicting damage upon germ cells, attributable to their minuscule size [31]. Fan et al. demonstrated that Ni NPs can penetrate the blood–testis barrier and infiltrate the spermatogenic tubule environment, adversely affecting spermatogenesis and the quality of sperm [32]. It has been found that nickel can accumulate in the testes of the armyworm [33]. Moreover, a distinction in reproductive toxicity has been observed, with nanoscale nickel exhibiting a more pronounced toxicity profile compared to nickel chloride and micron-sized nickel in vivo [34]**.** These observations suggest that the particulate nature of Ni NPs may be predominantly responsible for their toxicological impact. Nonetheless, definitive evidence confirming the translocation of Ni NPs across the blood–testis barrier is lacking, implying that the observed toxicity may be indirectly related, potentially mediated by nickel ions liberated from the nanoparticles.

Herein, Ni NPs induced the overproduction of ROS and decreased the ATP content and MMP levels in GC-1 cells, which is consistent with a previous study reported by Li et al. [35]. Ni NPs can cause mitochondrial dysfunction according to the Western blot results. Mitochondrial dynamics may have a significant impact on the process of excessive cell apoptosis induced by Ni NPs through a mitochondria-mediated intrinsic pathway. The dysregulation of mitochondrial division/fusion is associated with energy deficits, emphasizing the close relationship between the mitochondrial structure and function [36]. By adding the inhibitor Mdivi-1 to intervene, we explored and verified the regulation of mitochondrial division from the perspective of drug therapy. Then, from the perspective of gene therapy, the regulatory mechanism of mitochondrial division during the reproductive toxicity of Ni NPs was discussed. Two-way validation studies showed that the low expression of mitochondrial division inhibitors Mdivi-1 and Drp1 can indeed inhibit mitochondrial division.

However, we found that neither the drug treatment of Mdivi-1 nor the low expression of Dnm11 lentiviral gene therapy alleviated the low expression of mitochondrial fusion proteins Opa1, Mfn1, and Mfn2 caused by Ni NPs in germ cells. This seems to imply that mitochondrial fusion does not have an obvious regulatory function in the process of excessive apoptosis induced by Ni NPs. This result coincides with the study of Miao et al. [37]. However, it has also been shown that although Mfn1 and Mfn2 have different roles in regulating spermatogonia differentiation, their deficiency impairs mitochondrial function and disrupts male fertility [38]. Therefore, the importance of mitochondrial fusion in Ni NP-induced male reproductive toxicity remains an open question.

While our in vitro study utilizing cultured mouse germ cells has provided valuable insights into the effects of Ni NPs on mitochondrial dynamics and subsequent reproductive toxicity, it is crucial to acknowledge the limitations of this system when extrapolating our findings to in vivo conditions and human applications. The controlled environment of in vitro cell cultures may not fully capture the complexity of the physiological processes that occur within a living organism.

In short, it is an indisputable fact that Ni NPs damage reproductive function, but this area of research is in its early stages, especially regarding research on the effect of Ni NPs on male reproductive function, which is still in its infancy. In vivo studies are necessary to validate and expand upon our in vitro observations, and such research will be critical in developing effective strategies for risk assessment regarding Ni NP-related reproductive toxicity. In an industrial society with an increasing incidence of infertility, in-depth research on the mechanisms behind reproductive toxicity and the detection of widely used nanomaterials, and the establishment of an effective prevention system, will be of great importance in relation to human reproduction.

## 5. Conclusions

Taken together, the results of our study demonstrate the ability to partially reverse the toxic effects of Ni NPs using Mdivi-1 and low Dnm1l expression, underscoring the potential for further investigation into the role of mitochondrial dynamics in the toxicity induced by Ni NPs. These findings suggest that mitochondrial division and fusion play important roles in regulating Ni NP-induced cytotoxicity in GC-1 cells. Our study provides new insights into the mechanisms underlying the toxic effects of Ni NPs on male reproductive function and suggests potential targets for intervention. Ultimately, this research may contribute to the protection of the ecological environment and the promotion of human health.

## Data Availability

The data that support the findings of this study are available from the corresponding author upon reasonable request.
